# Current Trends in Sensors Based on Conducting Polymer Nanomaterials

**DOI:** 10.3390/nano3030524

**Published:** 2013-08-27

**Authors:** Hyeonseok Yoon

**Affiliations:** 1 Alan G. MacDiarmid Energy Research Institute, Department of Polymer and Fiber System Engineering, Chonnam National University, 77 Yongbong-ro, Buk-gu, Gwangju 500-757, Korea; E-Mail: hyoon@chonnam.ac.kr; Tel.: +82-62-530-1778; Fax: +82-62-530-1779; 2 Department of Polymer Engineering, Graduate School, Chonnam National University, 77 Yongbong-ro, Buk-gu, Gwangju 500-757, Korea

**Keywords:** conducting polymers, nanomaterials, chemical sensors, biosensors, polypyrrole, polyaniline, poly(3,4-ethylenedioxythiophene)

## Abstract

Conducting polymers represent an important class of functional organic materials for next-generation electronic and optical devices. Advances in nanotechnology allow for the fabrication of various conducting polymer nanomaterials through synthesis methods such as solid-phase template synthesis, molecular template synthesis, and template-free synthesis. Nanostructured conducting polymers featuring high surface area, small dimensions, and unique physical properties have been widely used to build various sensor devices. Many remarkable examples have been reported over the past decade. The enhanced sensitivity of conducting polymer nanomaterials toward various chemical/biological species and external stimuli has made them ideal candidates for incorporation into the design of sensors. However, the selectivity and stability still leave room for improvement.

## 1. Introduction

Conducting polymers have found a wide range of applications in the various fields of electronics, optics, energy devices, medicine, actuators, and composites as a viable alternative to metallic or inorganic semiconductor counterparts. In particular, there has recently been huge demand for developing flexible or wearable electronics, displays, and other devices, in which conducting polymers can ultimately be used as true flexible organic conductors or semiconductors. The most notable property of conducting polymers is their inherent electrical conductivity, which is closely connected to the charge transfer rate and electrochemical redox efficiency. Most conducting polymers act as semiconductors in terms of conductivity although several studies on metallic conducting polymers have been reported. Unlike their inorganic counterparts, a weak intermolecular overlap of electronic orbitals combined with a greater degree of disorder in conducting polymers result in narrow electronic bands and a low mobility of charge carriers. Consequently, conducting polymers have had serious limitations in specific applications such as transistors and memories. For example, the performance of field-effect transistors (FETs) based on conducting polymers cannot rival that of FETs based on single-crystalline inorganic semiconductors, such as Si and Ge, which have charge carrier mobilities that are about three orders of magnitude higher [1]. The mobilities of FETs based on solution-processed conducting polymers [e.g., poly(3-hexylthiophene)] are generally found to be in the range of 0.1 cm^2^ V^−1^ s^−1^, similar to those of amorphous silicon FETs [2]. A mobility of 10.5 cm^2^ V^−1^ s^−1^ has been the best reported thus far [3]. Conducting polymer FETs are not practically suitable for use in applications requiring high switching speeds.

Another example of conducting polymer applications involves electrochromic devices for smart windows and flexible displays. Conducting polymers are one of the most attractive electrochromic materials because of advantages such as high coloration efficiency, rapid switching ability, and diverse colors [4]. The switching time is one of the important parameters in display technology. The electrochromism of conducting polymers is based on reversible redox reactions accompanying ion exchange, and is completely different from the operating mechanism of FETs. The switching time is mainly affected by redox reaction efficiency, which depends on the ion diffusion rate and conductivity. Notably, it was recently reported that poly(3,4-ethylenedioxythiophene) (PEDOT) nanotubes with wall thicknesses of 10–20 nm exhibited fast switching speeds of less than 10 ms, though the color contrast was low [5]. However, electrochromic displays based on conducting polymers have not yet been considered for commercialization. Stability, rapid response times, and efficient color changes are still critical parameters that need improvement.

Conducting polymers have also been used for sensor applications as a signal transducer. There are several important parameters in sensor technology, such as sensitivity, selectivity, and response time [6,7]. In most cases, a response time on the order of seconds is enough for human recognition. Thus, it is easier to meet the requirements for response time than for other parameters. The sensing mechanisms of conducting polymers can involve redox reactions, ion adsorption and desorption, volume and weight changes, chain conformational changes, or charge transfer and screening. Compared to the inorganic counterparts, conducting polymers have an advantage in achieving high sensitivity and selectivity by virtue of their chemical and structural diversity. Conducting polymers also share the strengths of polymers over other materials, including low-temperature synthesis and processing, large-area manufacture, flexibility, and cost effectiveness. As a result, conducting polymers can be competitive in sensor applications and sensors are therefore considered to be one of the most practical applications of conducting polymers.

Conducting polymers have mostly been synthesized in the form of powder and film using chemical and electrochemical polymerization methods, respectively. It is important to precisely control the structure and morphology of conducting polymers during the synthesis process, since most of them are insoluble in common solvents and not thermoplastic. There have been remarkable advances in the synthesis of nanomaterials over the past several decades. Particularly, a variety of metallic and inorganic semiconductor nanostructures have been fabricated, which have exhibited unique electrical, optical, and chemical properties. Nanostructured materials feature high surface-to-volume ratio and small dimensions, which are very beneficial for sensor applications. The high surface area facilitates enhanced interaction between the materials and analytes, which leads to high sensitivity, and the small dimensions enable fast adsorption/desorption kinetics for analytes in the material, which allows a rapid response time. Accordingly, recent years have witnessed a shift in sensor technology towards more sensitive recognition elements, highly sophisticated architectures, and miniaturization due to the emergence of nanomaterials and nanotechnology. Conducting polymer nanomaterials also have strong potential for yielding enhanced sensor performance compared to their bulk counterparts [8]. However, polymers are unstable at the nanometer scale due to the nature of covalent bonds, which makes their nanostructures unstable as well. Because of this, progress in the synthesis of polymer nanomaterials has been relatively slow so far, and only limited research has been conducted on what fascinating properties polymer nanomaterials possess, in contrast to inorganic nanomaterials [9].

There are several conducting polymers that have been extensively investigated for practical applications, which are summarized in Table 1. All of them possess high conductivity and good environmental stability, and their polymerization reactions are not only straightforward but also proceed with high yield. Polypyrrole (PPy) features low oxidation potential and good biocompatibility, and the advantages of polyaniline (PANI) include that the monomer is very inexpensive. Polythiophene (PTh) has many useful derivatives, one of which is PEDOT developed by Bayer AG (Leverkusen, Germany). PEDOT features optical transparency and can be soluble in water with polystyrene sulfonate (PSS). Owing to these desirable characteristics, PPy, PANI, PTh and their derivatives have become leading materials in various applications fields. This review addresses research efforts to fabricate and manipulate nanostructures mainly consisting of the representative conducting polymers for sensor applications, and highlights remarkable recent examples with a focus on materials functionalization, transduction mechanisms, and device characteristics in sensor applications.

**Table 1:** Representative conducting polymers.

Name	Structure
Polypyrrole (PPy)	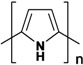
Polyaniline (PANI)	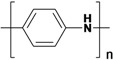
Polythiophene (PTh)	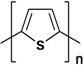
Poly(3,4-ethylenedioxythiophene) (PEDOT)	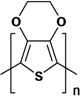

## 2. Fabrication of Conducting Polymer Nanomaterials

Precise control over the size and morphology of conducting polymers at the nanoscale is essential to improving the performance of related sensors. As mentioned, polymers are highly unstable at the nanometer scale, which is one of the greatest obstacles in building polymer nanoarchitectures. Nevertheless, numerous efforts have been made to fabricate polymer nanomaterials with well-defined size and morphology, and various types of conducting polymer nanostructures have been fabricated in a controlled fashion.

Conducting polymers have traditionally been synthesized via chemical or electrochemical oxidation polymerization. The overall process includes the oxidation of monomers, followed by the coupling of the charged monomers to produce the polymer chains. Chemical polymerization is advantageous for large-scale production at low cost, while electrochemical polymerization offers the possibility of in-situ formation, such as on an electrode for a sensor device. Conducting polymers can be obtained in the presence of various oxidizing agents. Oxidation polymerizations with acid or peroxide initiators result in insulating materials that require a post-doping process. Metal salts that can act as both oxidizing and doping agents are used to conduct the oxidation polymerization, which directly yields polymers in a conductive state. For example, ferric salts including FeCl_3_ and Fe(ClO_4_)_3_ are widely employed. Owing to their electrical conductivity, conducting polymers can grow electrochemically without oxidizing agents on an electrode. It is possible to tailor the polymer thickness by controlling the applied potential, polymerization time, and electrolyte.

Conducting polymer nanostructures have been fabricated with the aid of templates during the polymerization process. The synthetic routes are traditionally classified into three classes depending on the kind of template: hard template synthesis, soft template synthesis, and template-free synthesis. However, it is sometimes ambiguous to distinguish the hard and soft templates. For example, track-etched polymer membranes and polymer nanofibers are both soft, but they are categorized as a hard template and a soft template, respectively. Therefore, the templates are classified here as solid-phase and molecular templates as a new standard. Table 2 summarizes the templates frequently used for fabricating nanostructures and the possible resulting products.

**Table 2:** Template synthesis methods.

Products	Templates			
	Solid-Phase	Ref.	Molecular	Ref.
Nanospheres Nanocapsules	Nanospheres 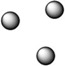	[10] [11,12]	Spherical micelles 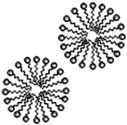	[13] [14]
Nanorods/fibers Nanotubes	Porous matrices 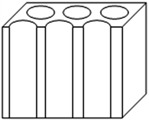	[15,16,17] [18,19]	Rod-like micelles 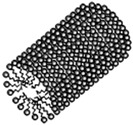	[20,21] [22,23]
Nanofibers Nanotubes	Nanorods 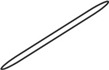	[24,25] [26]	Liquid crystals 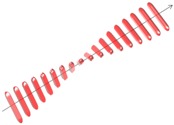	[27,28,29]

### 2.1. Solid-Phase Template Synthesis

Organic/inorganic nanoparticles, anodic alumina membranes, track-etched polymer membranes, and mesoporous silica are examples of solid-phase templates. Anodic alumina and track-etched polymer membranes with parallel cylindrical nanopores have often been chosen for the production of nanotubes or nanorods [30]. The porous alumina membranes are formed by anodizing high-purity alumina disks in an acidic electrolyte, and the track-etched polymer membranes are produced by irradiating the membrane with high-energy heavy ions, followed by chemical etching. The use of such solid-phase templates is advantageous for simply tailoring the dimensions of nanomaterials, and it has thus been extensively studied for synthetic routes to obtain 1D nanostructures of organic and inorganic materials. Conventional electrodeposition and electrophoresis techniques can be simply applied to the templates to yield nanostructures. Because the geometry and morphology of the resulting nanomaterials are endowed by the template itself, precise control of the diameter and length is possible with the solid-phase templates. However, it is very hard to completely remove the template without degradation or irreversible aggregation of the resulting nanomaterials. Scale-up for commercial applications is also highly difficult due to the complicated synthetic process and high cost. Therefore, the solid-phase template synthesis might be suitable for fabricating high-value products in the form of composites without the removal of template. A good example was suggested by Sommerdijk *et al*. [31]. A glucose sensor was developed using a track-etched membrane, in which PEDOT was coated onto the inner walls of the cylindrical pores. Since the PEDOT nanotube-containing membrane was easy to handle, it could be directly integrated into a sensing cell as a working electrode to detect glucose. This approach fully utilized the structural advantages of the PEDOT nanotubes in a sensor application, and thus offers a model for a successful way of using solid-phase templates.

**Figure 1:**
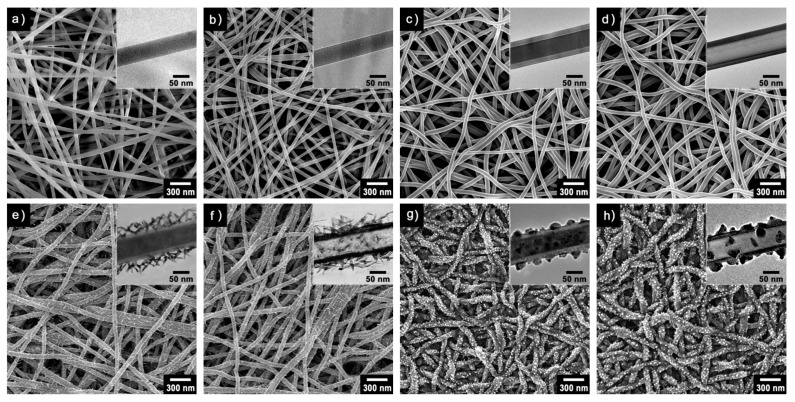
Multidimensional poly(3,4-ethylenedioxythiophene) (PEDOT) nanostructures with unique surface substructures. (**a**–**h**) The poly(methyl methacrylate) (PMMA) nanofibers function as a template and substrate for the growth of PEDOT under different synthetic conditions (temperature and pressure). The morphologies of the resulting nanomaterials were characterized by scanning electron microscopy (SEM) and transmission electron microscopy (TEM) (right top inset images): PMMA nanofibers (**a**) before and (**b**) after ferric ion adsorption; PMMA/PEDOT nanofibers with smooth layer surface (**c**) before and (**d**) after core etching; PMMA/PEDOT nanofibers with nanorod surface (**e**) before and (**f**) after core etching; PMMA/ PEDOT nanofibers with nanonodule surface (**g**) before and (**h**) after core etching. With permission from [33]; Copyright 2012, American Chemical Society.

Electrospinning is a simple and scalable technique that can create continuous and aligned polymer nanofibers and microfibers under a high electric field. Electrospun nanofibers are made from various polymer melts and solutions, which can serve as templates to direct the formation of conducting polymer nanofibers and nanotubes [32]. In a recent study [33], ultrathin poly(methyl methacrylate) (PMMA) nanofibers with an average diameter of 60 nm were obtained through electrospinning for use as a template (Figure 1a), and then immersed in ferric chloride solution for the adsorption of ferric ions on the PMMA nanofibers (Figure 1b). Subsequently, PEDOT was coated onto the PMMA nanofibers via vapor deposition polymerization at controlled temperatures and pressures to yield core-shell structured nanofibers (Figure 1c,e,g). Unique surface substructures such as nanonodules and nanorods could be grown on the nanofibers by adjusting major kinetic factors. The removal of the PMMA core can be done by selective solvent etching, which results in nanotubular structures (Figure 1d,f,h). The multidimensional nanofibers and nanotubes with surface substructures will have significant advantages when used as transducers for sensors.

### 2.2. Molecular Template Synthesis

**Figure 2:**
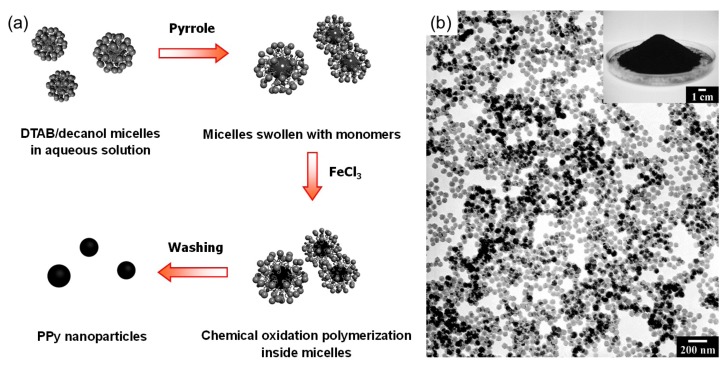
(**a**) Schematic illustration of the preparation of PPy nanoparticles in a cationic surfactant (DTAB)/co-surfactant (decanol) emulsion system; (**b**) TEM image of monodisperse PPy nanoparticles prepared through micelle templating (inset: photograph showing a Petri dish containing 12 g of PPy nanoparticles obtained in a single polymerization reaction). With permission from [13]; Copyright 2005, Wiley-VCH Verlag GmbH & Co. KGaA.

Molecular template synthesis has strengths compared to solid-phase template synthesis. The most widely used molecular templates include surfactants, liquid crystals, and polyelectrolytes. Because molecular template synthesis is comparatively straightforward and cost-effective, it is suitable for large-scale production. However, the molecular templates are not robust, static entities, leading to considerable difficulty in obtaining the desired nanostructures. Surfactant templating is a typical example of molecular template synthesis. Surfactants have the ability to self-assemble into ordered molecular structures called micelles. The micelles exist in various forms, and their dimensions generally range from a few to a few tens of nanometers, making them very suitable as templates. Microemulsions consisting of thermodynamically stable micelles with size less than 50 nm have been used in polymerization techniques to prepare polymer nanoparticles. However, micelles are highly sensitive to the surrounding environment, which makes it very difficult to achieve stable microemulsion systems for polymerization, preventing their widespread utilization for industrial products. One of the strategies for making stable microemulsion systems is to use co-surfactants like long-chain alcohols. A notable example is described in Figure 2a [13]. A cationic surfactant, dodecyltrimethylammonium bromide (DTAB) was used to obtain spherical micelles reinforced with decanol in aqueous solution. The decanol can play a role in retarding the diffusion of monomers through the aqueous phase. Consequently, monodisperse PPy nanoparticles (60 nm in diameter) were obtained on a gram scale, which is a very large quantity in laboratory-scale synthesis. Figure 2b shows a TEM image of the PPy nanoparticles and the inset shows a photo of a Petri dish containing 12 g of the nanoparticles.

**Figure 3:**
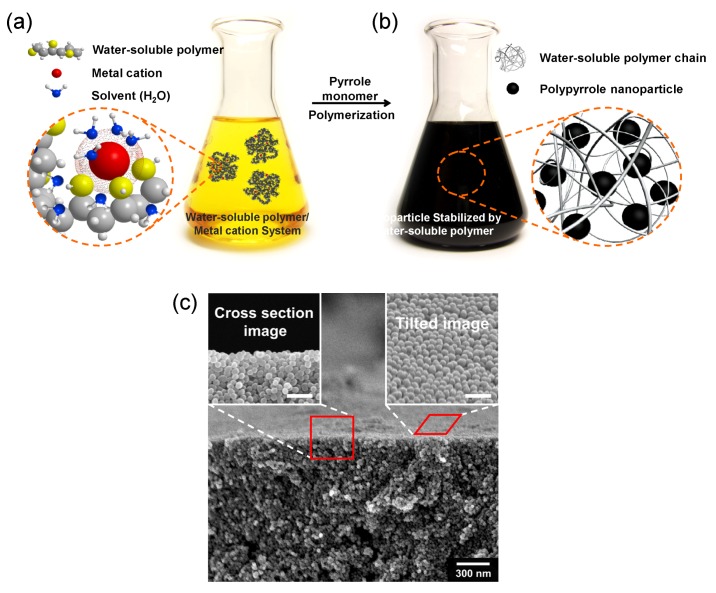
Schematic illustration of the formation of PPy nanoparticles in an aqueous dispersion of water-soluble polymer (PVA)/metal cation (ferric ion) complexes, and SEM images of the resulting nanoparticles. (**a**) Hydroxyl groups of PVA chains coordinate with ferric ions by an ion-dipole interaction in an aqueous medium; (**b**) The ferric ions act as the oxidizing agent for chemical oxidation of pyrrole monomers. After polymerization, the resulting PPy nanoparticles are stabilized by PVA chains; (**c**) Tilted and cross-section SEM images of the PPy nanoparticles stacked on a substrate (scale bar: 100 nm). With permission from [34]; Copyright 2010, Wiley-VCH Verlag GmbH & Co. KGaA.

This example indicates potential for use of the microemulsion technique for the efficient mass production of nanoparticles. However, the technique requires high surfactant concentration, which is problematic in terms of cost and environmental pollution. Another study was reported for producing PPy nanoparticles using a kind of dispersion polymerization, where water-soluble polymers provide steric stability. It is known that dispersion polymerization produces polymer microbeads. Jang *et al*. were able to produce PPy nanoparticles just by using a polymeric stabilizer, polyvinyl alcohol (PVA), in aqueous solution [34]. Figure 3a,b represent the synthesis process in which ferric ions were anchored on PVA chains via coordination bonding. The polymerization of pyrrole monomers proceeds only with ferric ions on PVA chains, which allows enough steric stability to yield nanometer-sized particles, as illustrated in Figure 3b. Figure 3c exhibits the SEM images of the resulting PPy nanoparticles, in which the size of the nanoparticles was surprisingly as small as *ca.* 25 nm in diameter. Considering that previous studies were limited to the synthesis of micrometer-sized particles with steric stabilizers, this approach is very innovative.

**Figure 4:**
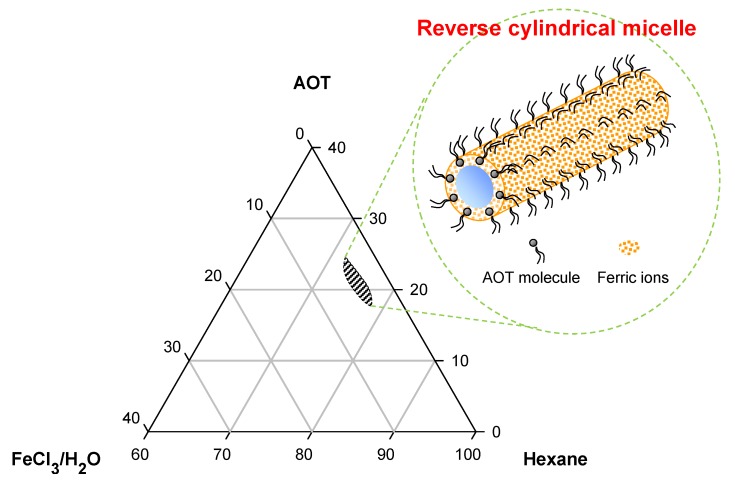
Partial ternary phase diagram for the hexane/AOT/aqueous FeCl_3_ solution system determined at 15 °C. The molar concentration of the aqueous FeCl_3_ solution was 16.2 M. The marked area corresponds to the AOT reverse cylindrical micelle phase region. With permission from [22]; Copyright 2005, American Chemical Society.

As shown in these examples, many examples of the formation of spherical nanoparticles can be found. However, it is hard to fabricate one-dimensional (1D) nanostructures such as nanofibers and nanotubes using molecular templates, and only a few studies have been reported [35]. 1D nanostructures have advantages over spherical or film-type counterparts in sensor applications, such as anisotropic physical properties and easy integration with two-terminal microelectrodes. Jang and Yoon reported the formation of PPy nanotubes in a reverse micelle system, which was the first notable example of fabricating conducting polymer nanotubes with a molecular template [22,23]. Sodium bis(2-ethylhexyl) sulfosuccinate (AOT) molecules were assembled with ferric ions to reverse cylindrical micelles in an apolar solvent, where ferric ions were adsorbed on anionic AOT headgroups. Figure 4 shows a partial ternary phase diagram of a hexane/AOT/aqueous ferric chloride solution. AOT has hydrophobic twin tail groups, and its sulfonate headgroup forms hydrogen bonds with water, making the AOT reverse micelles highly stable in an oil phase. Pyrrole is hydrophobic. Therefore, the oxidation polymerization of pyrrole monomers proceeds by ferric ions onto the reverse cylindrical micelles, leading to 1D nanostructures with hollow interiors.

Several different types of PPy nanowires have been obtained using surfactants, although their formation mechanisms have not been clarified. Hence, PPy has been a focus of many studies regarding the preparation of nanostructures. PPy can have a cross-linked molecular structure during polymerization, which would enhance its dimensional stability at the nanometer scale and make it possible to form relatively stable nanostructures. PANI and PEDOT nanostructures have been also prepared with the assistance of surfactants. PANI nanowire networks were synthesized using a cationic surfactant, hexadecyltrimethylammonium bromide, and an organic diacid, oxalic acid, in aqueous solution. The PANI nanowires had diameters of 35–100 nm depending on the polymerization conditions [36]. Unique clip-like nanostructures of PPy, PANI, and PEDOT could be even fabricated in bulk quantities using an anionic oxidant/cationic surfactant complex as a template [37]. The judicious combination of main parameters, such as surfactants, oxidizing/doping agents, pH, temperature, and other structure-directing agents, provides infinite possibilities of fabricating nanostructures with desirable morphology.

### 2.3. Template-Free Synthesis

Template-free synthesis, which is based on the self-assembly of molecular building blocks, is naturally very straightforward. However, extensive efforts are required to design and synthesize building blocks that are able to assemble into nanostructures under certain conditions. Fortunately, several species (precursors) can spontaneously form nanostructures without such artificial efforts. Typically, PANI has intrinsically 1D morphology at the nanometer scale when borne in aqueous solution, and thus, it has been intensively investigated to fabricate 1D PANI nanostructures in the absence of templates [38,39]. The formation of PANI nanofibrils has been studied extensively. The growth control of PANI nanofibrils in the presence of a steric stabilizer was reported, in addition to their morphology-dependent electrochemical properties [40]. Wan *et al*. have actively reported the formation of PANI nanotubes under various synthesis conditions without templates [41,42,43]. It was found that subtle changes in polymerization parameters often result in drastic differences in the morphology of the resulting PANI product.

**Figure 5:**
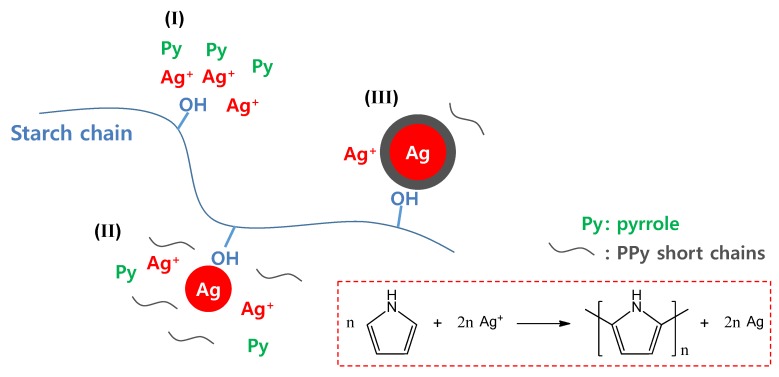
Schematic illustration of the formation mechanism of Ag-PPy nanoparticles: the reaction process could be divided into three stages (I, II, and III). Right bottom: the scheme describing the chemical reaction between pyrrole and silver cation. With permission from [47]; Copyright 2012, American Chemical Society.

Tseng *et al*. developed a site-specific electrochemical method for the fabrication of individually addressable PPy, PANI, and PEDOT nanowire frameworks on microelectrode junctions [44,45]. Numerous intercrossing polymer nanowire (50 to 200 nm in diameter) networks were formed between the two microelectrodes (2-μm gap). Shi *et al*. simply synthesized Au-PEDOT core-shell nanocables by the one-step interfacial polymerization of EDOT (in organic phase) and HAuCl_4_ (in aqueous phase) [46]. The nanocables have lengths of a few micrometers, outer diameters of around 50 nm, and central cores around 30 nm. The dissolution of the Au core using iodine solution gave rise to hollow PEDOT nanotubes. Similar research was also conducted to synthesize Ag-PPy core-shell nanoparticles with an average core diameter of 36 nm and a shell thickness of 13 nm via a simple one-pot synthesis, as represented in Figure 5 [47]. A silver precursor, silver nitrate, acted as an oxidizing agent for the chemical polymerization of pyrrole. The pyrrole monomers were oxidized by silver cations, yielding PPy and silver atoms concurrently (see the reaction scheme in Figure 5). In the initial stages I and II, the hydroxyl groups of soluble starch provide nucleation sites where silver cations are reduced to atomic silver by the pyrrole monomers, whereas pyrrole monomers are oxidized to radical cations that lead to the generation of PPy short chains. While the silver nanoseeds are being formed in stage II, additional silver cations are adsorbed onto the nanoseed surface due to a sort of common ion adsorption effect. The silver nanoseed surfaces become the active sites for oxidation of the surrounding pyrrole monomers and PPy short chains, finally resulting in the formation of a PPy shell. These bottom-up approaches offer an efficient and simple route for the fabrication of a nanostructured metal/conducting polymer complex.

## 3. Sensor Applications

A variety of sensors have been formulated using conducting polymers in different transduction modes. The transduction modes can be divided into five main classes based upon the operating principle into *conductometric*, *potentiometric*, *amperometric*, *colorimetric*, and *gravimetric* modes [48,49]. The conductometric mode uses changes in electrical conductivity in response to an analyte interaction. The conductivity of a conducting polymer material bridging the gap between two adjacent electrodes is commonly measured as a function of analyte concentration, and it can be also monitored with a fixed potential in solution.

Potentiometric sensing mode is based on analyte-induced changes in the chemical potential of a system when no current is flowing. The change in the open-circuit potential of the system is monitored, which is mostly proportional to the logarithm of the concentration of analyte. The chemical and diffusion processes have to be at equilibrium conditions in the potentiometric mode for a thermodynamically accurate signal to yield.

Amperometric mode refers to either single-potential amperometry or variable-potential amperometry. The principle of amperometric sensing is to measure the current generated by the redox reaction of an analyte at a sensing (working) electrode, where the current is subject to Faraday’s law and a dynamic reaction achieving steady-state conditions in the system.

Voltammetric mode is also a variant of amperometric mode. It monitors the change in current while varying the applied potential. Colorimetric sensors quantitate changes in optical absorption characteristics, which depend on the local electronic structure. The sensitivity of the bandgap of conducting polymers to analyte-induced changes provides a useful means to create this kind of sensor.

Lastly, gravimetric mode takes advantage of a weight change in a conducting polymer as a result of analyte-polymer interaction. Minute weight changes in the polymer can be normally monitored using a quartz crystal microbalance. Numerous conducting polymer sensors based on these transduction mechanisms have been devised in order to detect various chemical and biological species.

### 3.1. Chemical Sensors

There are many chemical species of concern that must be detected, including toxic gases, volatile organic compounds, alcohol, and humidity. Various conducting polymer nanostructures have been used to detect them. Chemically synthesized polymer nanoparticles suspended in solvents can be simply deposited on a prefabricated electrode by drop casting to construct a sensor substrate. Kwon *et al*. investigated the gas sensing properties of PPy nanoparticles with diameters of 20, 60, and 100 nm. They found that decreasing the nanoparticle size produced a noticeable increase in the sensitivity toward ammonia gas. It was anticipated that the increased sensitivity can be attributed to the higher surface-to-volume ratio of the 20-nm nanoparticles compared to the 60 and 100-nm nanoparticles [50]. The use of conducting polymer nanoparticles obtained via micelle templating and electrospinning in sensor applications has also been reported. Highly sensitive chemiresistive sensors based on PPy nanotubes were devised to discriminate volatile organic compounds and toxic gases [51]. PPy nanotubes were transferred onto a polydimethylsiloxane substrate using a dry-transfer method and then micro-patterned gold electrodes were deposited onto the nanotubes by thermal evaporation. Other conducting polymer nanostructures such as PPy nanoparticles, PEDOT nanorods, and PEDOT nanotubes were also integrated into electrode substrates to induce discriminative responses toward individual analytes. The lowest detectable concentration of the sensors was *ca.* 0.01 ppm for ammonia, selectively.

**Figure 6:**
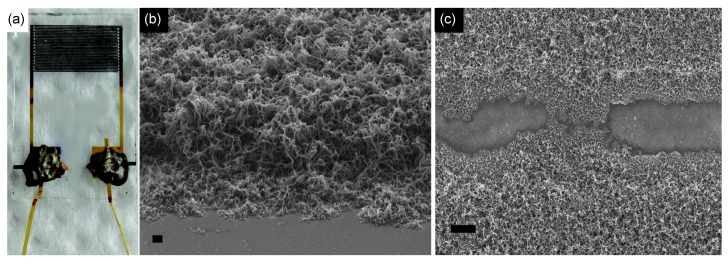
(**a**) Photograph of a developed gas sensor electrode. SEM images of PPy nanowires deposited on the electrode substrate: (**b**) 65° tilted view (scale bar = 1 μm) and (**c**) top view showing PPy nanowires bridging the insulating gap between the gold electrodes (scale bar = 10 μm). With permission from [52]; Copyright 2012, American Chemical Society.

Electrochemical polymerization allows controllable polymer deposition on an electrode. Recently, PPy nanowires grown electrochemically on a microelectrode substrate were used as conductometric transducer to detect hydrogen gas at room temperature (see Figure 6a) [52]. The diameters of the nanowires were in the range of 40–90 nm and their lengths were on the order of several tens of micrometers. It was found that the PPy nanowires were stacked at high density (Figure 6b), and a number of the nanowires bridged the gap between the electrodes (Figure 6c). The sensor was exposed to different concentrations of hydrogen gas at room temperature in a closed chamber, during which the resistance of the PPy nanowire electrode was monitored. Upon exposure to hydrogen gas, a drop in resistance was observed. The resistance of the PPy nanowires is expected to decrease when exposed to hydrogen, which is a reducing gas. The sensors had a linear detection range of *ca.* 600–2500 ppm for hydrogen gas, and their sensitivities were found to be dependent of the amount of the deposited nanowires. On the other hand, the sensors showed a loss in sensitivity due to carbon monoxide gas interference, which has remained a critical issue to be solved. The detection of hydrogen at low concentrations is very important, because it is explosive in the presence of oxygen. Interestingly, it has been found that PANI has the ability to interact with hydrogen. Kaner and Weiller demonstrated that hydrogen interacts directly with doped PANI nanofibers to induce a small change in the conductivity of the nanofibers [53]. The direct mass uptake of hydrogen by PANI nanofibers was also observed using a quartz crystal microbalance (*ca.* 3% relative to the nanofiber mass). A plausible mechanism of the hydrogen/PANI interaction involves hydrogen interacting with doped PANI at the charged amine nitrogen sites, followed by the dissociation of hydrogen with the formation of new N–H bonds at the amine nitrogen of the PANI chain [53,54]. Subsequently, charge transfer between adjacent amine nitrogens returns the PANI back to its original doped, emeraldine-salt form with a release of hydrogen.

**Figure 7:**
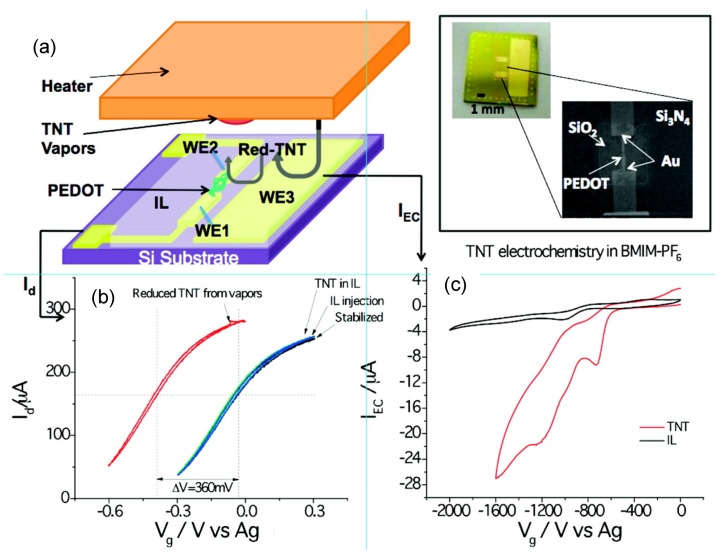
(**a**) A hybrid nanosensor consisting of a conducting polymer nanojunction (polymer bridged between WE1 and WE2) and a working electrode (WE3) on a Si chip. The chip is covered with a thin layer of ionic liquid (BMIM-PF_6_) serving as an electrolyte and preconcentration medium. Upon heating TNT particulates (to 60 °C), TNT vapor is generated which is collected by the ionic liquid layer. The analyte is reduced and detected electrochemically on WE3, and the reduction products are detected by the polymer nanojunctions. Inset: Optical micrograph of the sensor chip used and an SEM image of the PEDOT nanojunction; (**b**) Current (*I*_d_) via the PEDOT nanojunction plotted as a function of WE1 potential before and after exposure to TNT; (**c**) Cyclic voltammograms of a blank BMIM-PF_6_ solution (black) and 4 ppm TNT in BMIM-PF_6_ (red). The large reduction current in the latter case is due to the reduction of TNT. Note that an Ag wire quasi-reference electrode and a Pt counter electrode are used. With permission from [55]; Copyright 2010, American Chemical Society.

An integrated electrochemical and electrical nanosensor for the sensitive and selective detection of nitroaromatic explosives vapors (TNT) was demonstrated recently [55]. The integrated sensor chip consists of two main parts with a thin layer coating of BMIM-PF_6_: (i) PEDOT nanojunctions for conductometric detection and (ii) electrodes for electrochemical detection (Figure 7). The sensor utilizes both the electrochemical signatures of the reduction of the analytes, and the specific interactions of the reduction products with the polymer nanojunction to achieve excellent selectivity. BMIM-PF_6_ is used not only as a stable electrolyte, but also as a preconcentration medium for nitroaromatic compounds. Because TNT has a low vapor pressure (4.8 × 10^−6^ Torr) at room temperature, TNT residues are expected to last for a long time under ambient conditions, which makes it possible for them to be collected and detected in vapor form upon heating (Figure 7a). Upon exposure to the TNT vapor with no potential applied to WE3, the characteristic I_d_–V_g_ curve of the PEDOT nanojunction remains unchanged (cyan curve in Figure 7b). In contrast, when a negative enough potential (−1.8 to −1.2 V) is applied to the electrode, the cyclic voltammogram (red curve in Figure 7c) shows multiple peaks, due to the reduction of TNT. The total amount of the potential shift reached *ca.* −0.36 V within 2 min (red curve in Figure 7b), which provided sensitive detection of TNT. The sensor was capable of detecting ppt-level TNT within 1–2 min in the presence of various common interferents found in ambient air.

**Figure 8:**
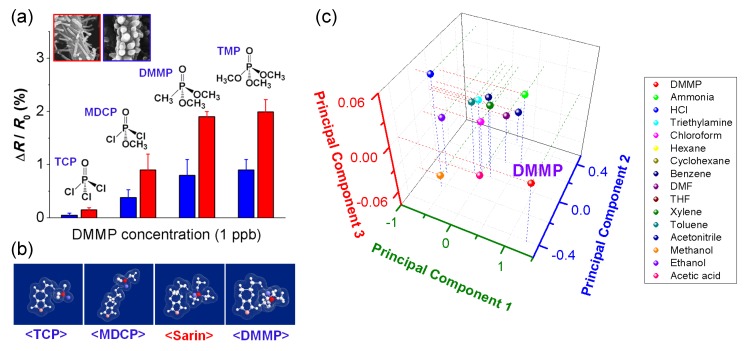
Sensing performance of chemical nerve agent sensor based on hydroxylated PEDOT nanotubes (HPNTs). (**a**) Histogram showing the response of HPNTs toward similar organophosphorus compounds at 1 ppb (TCP, MDCP, DMMP, TMP); (**b**) 3D graphics showing the formation of hydrogen bonds between nerve agent stimulant molecules and HEDOT; (**c**) Principal components analysis plot using response intensity inputs from four different conducting polymer nanomaterials (two different HPNTs, pristine PEDOT nanotubes, and PPy nanotubes) to the 16 analytes (including DMMP): each analyte concentration was fixed at around 4 ppm. With permission from [59]; Copyright 2012, American Chemical Society.

Conducting polymers can be complexed with many species to create composite materials that are sensitive to certain chemical agents. PANI nanofibers treated with CuCl_2_ showed the ability to detect hydrogen sulfide with change in resistance by 4 orders of magnitude [56]. The CuCl_2_ in the PANI nanofibers reacts with hydrogen sulfide to yield HCl, finally resulting in the further doping of the nanofibers. This reaction scheme has been extended to the detection of other specific analytes such as phosgene [57] and arsine [58]. Phosgene, a colorless toxic gas with a permissible exposure level of 0.1 ppm, was used as a chemical weapon during World War I. Arsine is also a highly toxic gas used in the semiconductor industry, with a permissible exposure level of 50 ppb. Water-soluble amines such as ethylenediamine, phenylenediamine, and metanilic acid were found to make PANI nanofibers sensitive to phosgene. In addition, water-soluble metal salts were be readily incorporated into PANI nanofibers, such as CuCl_2_, CuBr_2_, CuF_2_, Cu(O_2_CCH_3_)_2_, Cu(NO_3_)_2_, EuCl_2_, NiCl_2_, FeCl_3_, and CoCl_2_. The salts were screened to find the best candidates for detecting arsine. The CuBr_2_/PANI nanofiber composite gave the best response with a change in resistance of more than an order of magnitude upon exposure to arsine. One of the notorious toxic gases is sarin, which is an organophosphorous compound. Flexible nerve agent sensors based on hydroxylated PEDOT nanotubes (HPNTs) with unique surface substructures were recently demonstrated [59]. Axially aligned HPNTs were produced via electrospinning process under a magnetic field, and then integrated into a conductometric substrate. Dimethyl methylphosphonate (DMMP) is commonly used as a simulant for sarin. Upon exposure to DMMO, the HPNTs showed a sharp increase in resistance. The reaction time was as fast as *ca.* 1 s, and the recovery time was as good as 3–25 s. The lowest detectable concentration was as low as 10 ppt. Figure 8 provides information on the selectivity of the HPNTs toward similar organophosphorus compounds such as trimethyl phosphate (TMP), methyl dichlorophosphate (MDCP), and trichlorophosphate (TCP), used as nerve gas simulants. The hydroxyl side chain of PEDOT was found to interact with the nerve agent simulants through hydrogen bonding. The hydrogen-bond strength was calculated to increase in the order TCP < MDCP < sarin ≈ DMMP < TMP (Figure 8b), which would lead to different response intensities with the same tendency (Figure 8a). The response of HPNTs against 15 volatile organic compounds, chosen as potential interferents, was examined and compared to responses from other sensing materials using principal components analysis. The first three principal component scores are plotted in Figure 8c. Unique signatures of the analytes could be observed, denoted by their segregation into separate regions of the plot, allowing identification of individual analytes by their responses. Particularly, DMMP exhibited clearly differentiable components, which validated the selective recognition ability of HPNTs. The response of the HPNTs was found to be durable even under mechanical deformation, which provided the possibility of fabricating a wearable nerve agent sensor.

**Figure 9:**
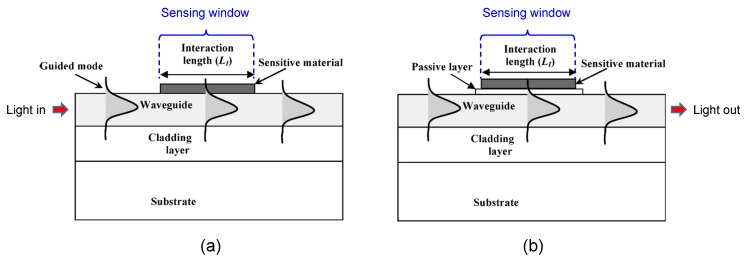
Schematic structures of the two types of waveguide sensors: (**a**) conventional waveguide sensor; (**b**) multilayer integrated waveguide sensor. With permission from [60]; Copyright 2008, American Chemical Society.

Optical modulation of the major properties of conducting polymers has offered attractive strategies for detecting various target species. Debarnot *et al*. recently developed a “multilayer integrated optical sensor (MIOS)” using PANI as a sensitive material and demonstrated the detection of ammonia gas [60]. Figure 9 schematically illustrates the structural difference between the conventional waveguide sensor and the MIOS. In the MIOS structure, a thin passive film of PMMA is deposited on a small section of the planar waveguide known as the interaction length (*L_I_*), and then the sensitive material PANI is coated onto the passive film. The planar waveguide consists of a glycidyl ether of bisphenol A, which is a promising candidate for simply fabricating optical waveguides due to the excellent optical transparency and easy processing [61]. PANI is opaque and has a high refractive index and absorption coefficient. PANI interacts with ammonia gas in such a way that the adsorption and desorption of ammonia give rise to deprotonation and protonation of PANI, respectively, leading to variation of the optical properties of PANI. The PMMA film is transparent with a refractive index lower than that of the waveguide to satisfy the total internal reflection condition for light propagation, and importantly, it reduces the optical losses induced by just evanescent wave/sensitive material coupling. A significant change was observed in the guided light output power after the sensor was exposed to ammonia gas, due to the variation in PANI absorption characteristics. Compared to the conventional transmission technique, the planar waveguide provides a much longer light propagation distance due to the multiple reflections of the guided light, leading to higher sensitivity. The sensor exhibited a logarithmic linear optical response within a range of ammonia concentration of *ca.* 90–4600 ppm.

### 3.2. Biosensors

**Figure 10:**
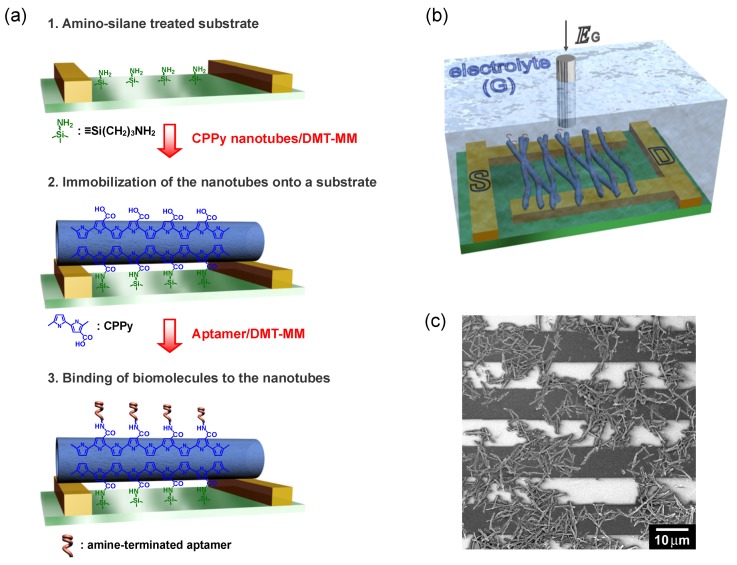
Schematic illustration of (**a**) reaction steps for the fabrication of a sensor platform based on carboxylated PPy nanotubes; and (**b**) a liquid ion-gated FET sensor; (**c**) SEM image of carboxylated PPy nanotubes that are deposited on the interdigitated microelectrode substrate. With permission from [62]; Copyright 2008, Wiley-VCH Verlag GmbH & Co. KGaA.

Biosensing is commonly conducted in a solution phase to maintain the biological activity of target species. Accordingly, it is critical to immobilize transducer materials on sensing platforms for continuously obtaining reliable signals. Compared to metals and ceramics, conducting polymers are more compatible with biological systems. Inorganic nanomaterials have been readily integrated into biosensor platforms using lithography and focused ion beam techniques. However, the integration of conducting polymer nanomaterials into biosensors has been limited due to their incompatibility with the traditional microfabrication processes. Chemical, thermal, and kinetic damage can possibly degrade conducting polymers during the microfabrication process. An alternative strategy was developed to circumvent this issue by Jang and Yoon [62]. Covalent linkages between PPy nanotubes and a microelectrode substrate were made to achieve reliable electrical contact in solution. PPy nanotubes with carboxyl groups were prepared and the carboxyl groups were chemically coupled with the surface amino group of the electrode substrate (Figure 10a). A liquid, ion-gated field-effect transistor (FET) sensor could be successfully fabricated using this method (Figure 10b,c). The structure of the FET sensor is similar to that of the normal metal-oxide-semiconductor FET, except for the gate, which incorporates the means of transduction from a chemical event to a voltage [63]. In the liquid ion-gated FET configuration, two metal electrodes, called the source and drain are deposited on a substrate. The gate electrode is electrically isolated from the two electrodes and immersed in an electrolyte, as illustrated in Figure 10b. The gate potential affects the density of charge carriers in the semiconductor channel. The channel is normally modified with molecular or polymeric receptors for selectively recognizing the analyte of interest. The recognition of the analyte by the receptor can alter the gate potential being applied on the channel, which modulates the source-drain current. With this operating principle, many different types of FET sensors have been devised in order to detect glucose [64], odorants [65], tastants [66], hormones [67], and proteins [68]. Several critical parameters determining the FET sensor response have been also investigated. For example, Mulchandani *et al*. fabricated single PPy nanowire-based FET sensors for real-time pH monitoring and examined how the diameter of the nanowire affects the sensor performance [69]. PPy nanowires with three different diameters (*ca.* 60, 80, and 200 nm) were anchored on a pair of gold electrodes with different gap lengths (1 and 4 μm). The FET sensors had higher sensitivity with lower diameter and higher length.

**Figure 11:**
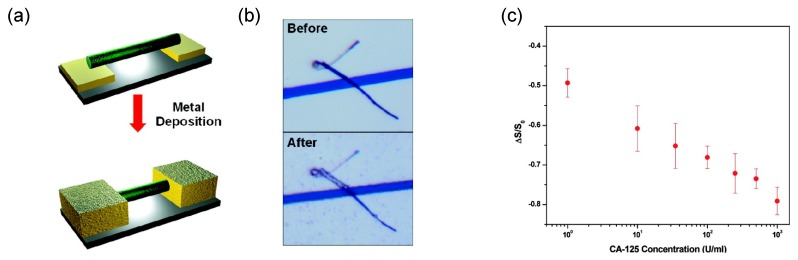
(**a**) Schematics of maskless electrodeposition of gold along with (**b**) optical images of before and after selective gold electrodeposition on gold microelectrodes separated by a 3 μm gap connected with a single PPy nanowire; (**c**) A calibration curve in terms of normalized conductance change of a single PPy nanowire biosensor in spiked human blood plasma suggesting the utility of this sensor for real sample measurements. With permission from [73]; Copyright 2009, American Chemical Society.

Electrochemical deposition can also be an excellent approach to fabricating biosensor platforms based on conducting polymer nanomaterials. Mulchandani and Myung reported a single-step electrodeposition technique for the formation of PPy and PANI nanowires to bridge the gap between two electrodes on a silicon wafer [70,71]. The deposition and growth of the nanowires are based on electrochemical oxidation polymerization. The nanowires grew from the cathode to the anode through an electrolyte channel when an electric potential was applied, and the dimensions of the nanowires were determined by the width of the channel and the distance between electrodes. Since this process is biomolecule-friendly, receptors can be introduced into the polymer during the electropolymerization. For example, avidin protein could be entrapped in the PPy nanowire, which allowed the detection of a biotin-conjugated DNA [72]. A similar electrochemical approach was developed by the same research group [73]. They used dielectrophoretic alignment to achieve a single PPy nanowire connection between a pair of microelectrodes, and then performed maskless selective gold electrodeposition on the microelectrodes to anchor the nanowire ends (Figure 11a,b). The nanowire surface with the secondary amine groups was functionalized with an antibody using carbodiimide linker chemistry to detect a cancer antigen. The detection of cancer antigen CA 125 in human blood plasma was demonstrated, showing the utility of the sensor for real samples (Figure 11c).

Another important issue in biosensors is related to how receptors are coupled with transducers. Versatile receptors including enzymes, antibodies, nucleic acids, and cells have been introduced into conducting polymers through adsorption, entrapment, or covalent binding. A widely known example involves enzyme-based glucose detection for diagnosing and managing diabetes. Traditionally, amperometric glucose detection has been achieved with glucose oxidase (GO*x*) enzyme electrodes. The GO*x* has been immobilized on electrodes by chemical cross-linking, eletrodeposition or electrostatic interactions [74]. Recent studies have proposed new alternatives to the conventional enzyme electrode [75,76]. Conducting polymer nanomaterials have mainly been employed as the conductive matrix with redox properties. A FET-based sensor was developed by using GO*x* enzyme-attached PPy nanotubes as the conductive channel [64]. The PPy nanotubes were prepared by the chemical polymerization of pyrrole-3-carboxylic acid inside the cylindrical pores of an alumina membrane. GOx contains terminal amino groups on its lysine residues. Thus, GO*x* can be covalently attached to the nanotubes via condensation reaction between the carboxylic group and the amino group. Cai *et al*. took advantage of the covalent attachment of GO*x* to PANI nanofibers for glucose detection [77]. They used a well-known EDC/NHS chemistry to attach the enzyme to the nanofibers. GO*x* kept its natural structure and electroactivity, and consequently, the nanofiber electrode showed a wide linear range of 0.01 to 1 mM with a detection limit of 0.5 μM for glucose. In contrast, a conducting polymer nanojunction sensor described by Tao *et al*. just used a simple adsorption of GO*x* on polyaniline/poly(acrylic acid) nanojunction to build up an enzyme electrode [78]. The nanojunction sensor showed specific and rapid responses toward glucose with the detection limit of micromolar levels. A new type of amperometric glucose sensor developed by Wu and Yin used PANI-wrapped boron nitride nanotubes decorated with Pt nanoparticles as the electrode material [79]. GO*x* was just mixed with the Pt/PANI/boron nitride nanotubes, which resulted in a cotton-like enzyme nano-hybrid. Surprisingly, the enzyme nano-hybrid electrode had good acid stability and heat resistance.

Molecularly imprinted sensors that require no receptors have also been fabricated using conducting polymers. For example, a simple method for the photoelectrochemical sensing of microcystin-LR (MC-LR) was achieved by using PPy/titania nanotubes with MC-LR recognition sites [80]. MC-LR is a chemical species that may cause structural and functional disturbances of the liver, and is a potential cancer threat [81]. Owing to its ubiquity and high toxicity, MC-LR is an element of concern in water-purity control and environmental monitoring. Optical transduction can facilitate the detection of electrochemically inert species such as MC-LR. Titania nanotubes have high surface area and photoelectric activity. The recognition sites of MC-LR were imprinted in the interior PPy layer of titania nanotubes. The photocatalytic oxidation of MC-LR under a certain bias potential caused changes in the photocurrent, and PPy played a role in further enhancing the photoelectric conversion efficiency. MC-LR could be determined with a linear range of 0.5 to 100 μg L^−1^, and the detection limit was 0.1 μg L^−1^. In another example, Kan *et al*. reported an electrochemical sensor based on molecularly imprinted PPy for epinephrine recognition [82]. Epinephrine, also known as adrenaline, is an important neurotransmitter in the mammalian central nervous system, and is present in human serum at nanomolar levels. Pyrrole monomers were electrochemically polymerized with epinephrine molecules on the surface of an electrode coated with silica nanoparticles and carbon nanotubes. Epinephrine can be imprinted inside PPy via hydrogen bonding during the polymerization. After the removal of silica nanoparticles and epinephrine, a molecularly imprinted PPy/carbon nanotube film with high porosity could be produced on the electrode. The amperometric response of the PPy/carbon nanotube electrode was recorded by the addition of epinephrine in phosphate buffer solution. Good linearity between the epinephrine concentration and the current response was observed in the range of 3 × 10^−7^ to 1 × 10^−3^ M and the detection limit was found to be 3 × 10^−8^ M.

### 3.3. Others

**Figure 12:**
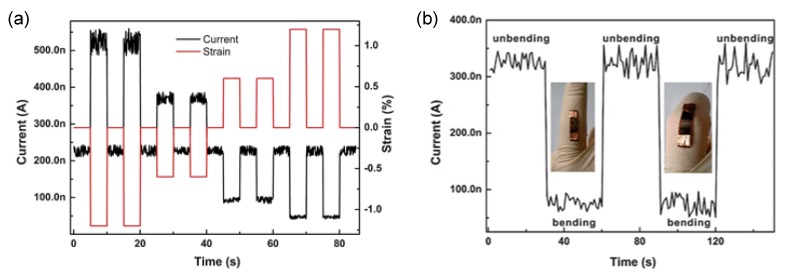
(**a**) The change in resistance of the strain sensor under different external strains; (**b**) The current response of the strain sensor with cyclical bending and unbending actions of the finger. With permission from [84]; Copyright 2011, Royal Chemical Society.

Mechanical sensors based on conducting polymers that transduce dynamic responses into measurable signals are receiving growing attention [83]. As a notable example, a strain sensor was developed by using electrospun PEDOT/PSS/PVA nanofibers [84]. PEDOT/PSS/PVA nanofibers were collected on a polyimide (Kapton) film with two patterned contact electrodes and then annealed at 80 °C for 15 min. The nanofiber film was packaged with a thin layer of polydimethylsiloxane to prevent contamination, and its current change was monitored under external strains. Figure 12a shows the time-resolved current response of the sensor to repeated exposure to different tensile strains. The current decreased with tensile strain, and increased with compressive strain. The current was fully recovered to the original state when the strain was relieved, indicating that the sensor had high reproducibility and good stability in response. Additionally, the behavior of the sensor in response to human actions was examined, as shown in Figure 12b. The sensor was affixed to an index finger, and the current response during bending and unbending actions were recorded. The sensor gave an immediate response to the actions of the finger and the current response was highly reversible and reproducible.

## 4. Summary and Outlook

A number of studies have demonstrated that conducting polymer nanomaterials are promising candidates for building state-of-the-art sensors, due to their unique advantages over other materials. The most important parameters that determine the sensor performance include response/recovery time, sensitivity, selectivity, and stability. A great deal of effort has been directed toward enhancing these parameters over the past decades. The response and recovery times and the sensitivity have experienced impressive improvements with great advances in nanotechnology. However, selectivity is still a challenge. Detecting target species in a complex environment remains a difficult task, and is hindering the widespread application of conducting polymer-based sensors. Most sensors have been demonstrated only under controlled laboratory conditions, while actual samples comprise a range of interferent chemicals and substances. A conducting polymer by itself lacks the specificity or selectivity toward target species, and thus, it is crucial to judiciously functionalize the polymer with appropriate receptors. Another concern is that conducting polymers may degrade over time, even in dry, oxygen-free environments. New efforts should be aimed at improving the stability of the sensor response.

Sensors play an ever-increasing role in environmental monitoring, medical diagnosis, industrial safety control, security, and so forth. Conducting polymer nanomaterials are believed to have much unexplored potential for sensor applications. Thus, future research into conducting-polymer nanomaterials-based sensors will offer great potential for the construction of next-generation sensor devices. In particular, it is anticipated that wearable or flexible high-performance sensors will be developed using conducting polymer nanomaterials in the near future.
